# Myocardial T1 mapping as a diagnostic tool in pediatric patients with a concern for cardiac disease

**DOI:** 10.1186/1532-429X-18-S1-P167

**Published:** 2016-01-27

**Authors:** Cory V Noel, Ramkumar Krishnamurthy, Rajesh Krishnamurthy

**Affiliations:** 1 Pediatric Cardiology, Baylor College of Medicine, Houston, TX USA; 2 Radiology, Texas Children's Hospital, Houston, TX USA

## Background

Myocardial tissue characterization with both native T1 mapping and T1 mapping following gadolinium based contrast agents (T1 enhanced) has emerged as an important asset of CMR imaging [1]. However, there is only minimal experience in pediatrics [2]. Native T1 has shown to be a marker of myocardial edema, and may play a role in pathologic states such as myocarditis [3]. T1 enhanced mapping has shown to be a useful biomarker for disease sates with diffuse fibrosis, such as hypertrophic cardiomyopathy (HCM), and is comparable to myocardial biopsy [4].

Purpose: To examine the effectiveness of CMR myocardial characterization by native T1 and T1 enhanced mapping in a heterogenous group of pediatric patients with signs concerning for cardiomyopathy or myocarditis.

## Methods

We reviewed our initial experience of an ongoing study with T1 mapping in 10 subjects (aged 15.4 ± 2.5 years). All patients underwent CMR due to signs suggestive of cardiac disease. Cohort included 4 patients with concern for myocarditis, 4 with concerns for HCM, and 2 with concern for myocardial ischemia. 3 of the acquisitions were made in 3T Acheiva^TM^, remainder used 1.5T Ingenia^TM^ scanner (Philips Healthcare, Best Netherlands).

Acquisition Protocol: All clinically indicated MRI sequences were performed, including delayed enhanced phase sensitive inversion recovery sequence, after injection of contrast (Magnevist^TM^). T1 mapping (modified Look Locker - MOLLI) sequence was performed prior to contrast injection and post contrast (15 minutes after contrast) injection. The MOLLI sequence had a bSSFP readout TR/TE/α = 3/1.5 ms/20°; A 5,5,3 inversion acquisition scheme was used, and acquisition time was 13 heart beats.

## Data Analysis

The T1 was measured by manually drawing a region of interest at both the interventricular septum and the free wall after exporting the data to a custom made Matlab^TM^ program. Pixels with a zero value - representing a noisy T1 fit were ignored from the calculations.

## Results

The native and enhanced T1 were calculated for all patients except 1 who had significant artifact in the free wall on the native T1 sequence. The 2 patients with concern for myocardial ischemia, and 1 patient with possible HCM were assessed on the 3T. As assessed on the 1.5T, the native T1 of the free wall in patients who were clinically treated for myocarditis (n = 3) was significantly higher than the native T1 in all other patients (n = 4), 1094 ± 38 ms vs. 986 ± 23 ms (p < 0.05). Likewise, the septal T1 enhanced of patients who were diagnosed with HCM (n = 3) was lower than the remaining patients (n = 4), 460 ± 16 ms vs. 533 ± 16 ms (p <0.05).

## Conclusions

Myocardial characterization may be an effective tool in pediatric patients with a potential diagnosis of cardiomyopathy or myocarditis. Within a heterogenous group of patients, the native T1 was able to distinguish myocarditis from other potential cardiac disorders, while the enhanced T1 was able to distinguish HCM.

## References

[1] J CMR. 16: 2-

[2] Circ Imaging. e002504-2

[3] JACC Imag. 8: 1-

[4] Radiology. 256: 3-

